# Online gambling’s moderators: how effective? Study protocol for a randomized controlled trial

**DOI:** 10.1186/s12889-015-1846-7

**Published:** 2015-05-30

**Authors:** Julie Caillon, Marie Grall-Bronnec, Jean-Benoit Hardouin, Jean-Luc Venisse, Gaelle Challet-Bouju

**Affiliations:** Clinical Investigation Unit BALANCED “BehaviorAL AddictioNs and ComplEx mood Disorders”, Addictology and Psychiatry Department, Nantes University Hospital, 85 rue de Saint Jacques, 44093 Nantes Cedex 1, France; EA 4275 SPHERE “Biostatistics, Pharmacoepidemiology and Human Sciences Research Team”, Medicine and Pharmaceutical Sciences Faculty, Nantes University, Nantes, France; Methodology and Biostatistics Unit, Nantes University Hospital, Nantes, France

**Keywords:** Gambling disorders, Problem gambling, Online gambling, Prevention, Internet-based protection, Moderators, Self-exclusion, Self-limitation, Bonus, Information

## Abstract

**Background:**

Online gambling has been legalized in France in 2010. Licenses are issued to gambling operators who demonstrate their ability to respect the legal framework (security, taxation, consumer protection, etc.). The preventive measures to protect vulnerable gamblers include an obligation to provide online gambling moderators. These moderators should allow gamblers to limit their bets, exclude themselves from the website for 7 days, and consult the balance of the gambler’s account at any time. However, there are only a few published reports of empirical research investigating the effectiveness of Internet-based protective measures implemented by French law. Moreover, no empirical research has yet studied the impact of bonuses on gambling behaviors.

**Methods/Design:**

This research is an experimental randomized controlled trial, risk prevention targeted. The research is divided into four sub-studies depending on the studied moderator: limiting bonuses, self-exclusion, self-limitation and information. The study sample consists of 485 volunteers. For each experimental condition and the control groups, the sample is composed of gamblers equally recruited from gamblers having preferences in each of the three major forms of games (lottery and scratch tickets, sports and horserace betting, and poker). For each form of gambling, the gamblers are recruited in order to obtain as many problem gamblers as non-problem gamblers. According to the randomization, the experimental session begins. The experimental session is a gambling situation on a computer in our research center. The gambler is invited to play on his favorite gambling site as usual, with his own gambler account and his own money. Data collected comprise sociodemographic characteristics, gambling habits, an interview about enjoyment and feeling out of control during the gambling session, moderator impact on gambling practice, statement of gambling parameters and questionnaires (BMIS, GRCS, CPGI, GACS). Moderator efficiency is assessed based on the two major characteristics of gambling behavior: money wagered and time spent in gambling.

**Discussion:**

The results of this research will be important to prevent online problem gambling and influence policy-makers.

**Trial registration number:**

NCT01789580. Registered 8 February 2013

## Background

If the majority of gamblers maintain a recreational and controlled practice, some of them will develop an excessive practice. Gambling disorder is defined in the Diagnostic and Statistical Manual of Mental Disorders, Fifth Edition (DSM-V) as a persistent and recurrent problematic gambling behaviour leading to clinically significant impairment or distress [1]. The first French prevalence study conducted in 2010 showed that 1.3 % of adults in the general population were problem gamblers [2]. By focusing solely on gamblers during the year, a proportion of 2.8 % of problem gamblers was found. Another study conducted in 2013 among online gamblers showed a prevalence of problem gambling for 17 % [3]. These figures are higher than those observed with traditional games.

Several features can explain the fact that online gambling is more addictive compared with offline gambling [4]. Game availability and accessibility are certainly the most important risk factors on the Internet. A gambler can access an almost unlimited supply of games on the Internet from home, 7 days a week and 24 h a day. Moreover, online gambling lets you gamble on multiple sites at once, thus increasing gambling frequency and decreasing the time between setting and gain. This promotes loss of control in gamblers. The particular method of payment related to Internet support (use of a credit card) can also promote a loss of awareness of financial losses. Anonymity is also part of the same phenomenon. Many players prefer to play online because they feel no judgment about their gambling behavior contrary to what they may experience in real places where they can be identified as patrons or “big” players. This contributes to disinhibition phenomenon [5] of gambling practice, as well as the comfort brought by online practice (no need to prepare to go out, can play everywhere, can associate with the consumption of alcohol or drugs …). Finally, some commercial techniques facilitate online gambling as bonuses. Bonuses are amounts of money deposited by gambling operators in gambling accounts at the time of registration (acquisition bonus) or later (retention bonus). They represent about 50 % of the marketing budget for gambling operators or about 62 million euros in 2013 [6].

In France, the Regulatory Authority for Online Gambling (ARJEL) is the administrative authority that oversees the application of the law and issues licenses to gambling operators who have demonstrated their ability to respect the legal framework (security, taxation, consumer protection, etc.). The preventive measures to protect vulnerable gamblers include an obligation to provide online gambling moderators. These moderators should allow gamblers to limit their bets, exclude themselves from the website for 7 days, and consult the instant balance of the gambler's account at any time. However, with no precise specification for implementing these moderators, gambling operators, who have mainly commercial interests, often provide minimum protection for gamblers.

Furthermore, there are only a few published empirical research studies investigating the effectiveness of Internet-based protection implemented by the ARJEL. So we also opted to consider relevant research pertaining to the effectiveness of land-based protection.

About self-exclusion, Ladouceur and his colleagues [7] found that the majority of self-excluders at land-based venues had significantly more control over their gambling behavior, and reported positive effects on their life (mood, social and family life, work). Similar findings were reported in others research work [8–10]. To our knowledge, no study evaluates the effectiveness of self-exclusion on Internet gambling.

The effectiveness of self-limitation on money spent was studied on the Internet, but only for sports betting. Nelson and his colleagues [11] demonstrated that self-limitation programs appear to be promising options for Internet gamblers at risk for gambling problems, but very few used them . Self-limitation appears to help gamblers reduce their betting activity (frequency of betting, bets per day, and total wagered). Time spent in gambling, not just money spent, appears to be an important indicator of gambling problems, according to the type of games, but that was not studied.

Concerning information about gambling risks, Monaghan and Blaszczynski [12] indicate that pop-up messages may be an appropriate mode of presentation for harm-minimization signs on Electronic Gaming Machines (EGMs). The study demonstrates that self-appraisal messages had the greatest reported impact on a gambler’s thoughts and behavior. At present, information for harm-minimization does not exist in Internet gambling. Finally, no empirical research has studied the impact of bonuses on gambling behavior.

In the light of the specific addictive characteristics of online gambling, it is essential to evaluate the effectiveness of existing means of protection and propose new ones in order to improve responsible gambling measures specific to online gambling and thus protect the most vulnerable gamblers.

The main objective is to assess the effectiveness of four types of gambling moderators: limiting bonuses, self-exclusion, self-limitation and information. Some of them are already proposed by law, but not evaluated, and others are not yet available.

The efficiency of the moderators is assessed depending on gambling type (games of chance without skill, such as lottery or scratch tickets, semi-skilled games of chance such as sports betting or horserace betting, and skilled games of chance such as poker [13]) and gambler status (problem gambler or no problem gambler), and based on the two major characteristics of gambling behavior: money wagered and time spent in gambling. The aim is to provide a system of protection based on the type of game and the status of the player.

## Method/Design

The proposed research is an experimental randomized controlled trial, risk prevention targeted. The study is divided into four sub-studies depending on the studied moderator: limiting bonuses, self-exclusion, self-limitation and information.

### Ethical approval

The participants were informed about the research and gave their written informed consent prior to their inclusion in the study. This study was approved by the French Research Ethics Committee (CPP) on January 8, 2013.

### Common methodology to all sub-studies

Media announcements (newspapers, radio, and websites) are used to recruit participants. In addition, we have subcontracted recruitment to survey institutes to obtain lists of potential participants. Recruitment began in March 2013 and will end in March 2017. Volunteers are asked to contact the research team by email, to obtain details of the study and arrange a telephone appointment to complete the pre-selection questionnaire. Inclusion criteria are: age 18 or older, currently gambling at least once during the past month on a website authorized by ARJEL, agreeing to give access to the gambling-account data and to be filmed during the experimental session. Exclusion criteria are: scoring 8 or more on the Canadian Problem Gambling Index (CPGI) [14], being actually under treatment for a gambling problem, being indebted, using psychoactive substances on the day of the experiment, participating in another clinical study during the week preceding the experiment, being pregnant, being under protection (guardianship or curatorship), having an history of psychosis or cognitive impairment, having a betting limit of less than 200 € - for the sub-study “limiting bonuses” (in order to allow randomization under any experimental condition) .

If they meet the eligibility criteria, and after providing informed consent, participants will have an interview prior to the experiment, to collect the following informations: sociodemographic data, gambling history and current gambling behavior, severity of cognitive distortions based on the Gambling Related Cognitions Scale (GRCS) [15], severity of gambling problems based on the CPGI [14], mood based on the Brief Mood Inspection Scale (BMIS) [16], feelings of loss of control, intensity of craving based on the Gambling Craving Scale (GACS) [17], gambling-account information and knowledge and use of online gambling protections.

**Fig. 1:**
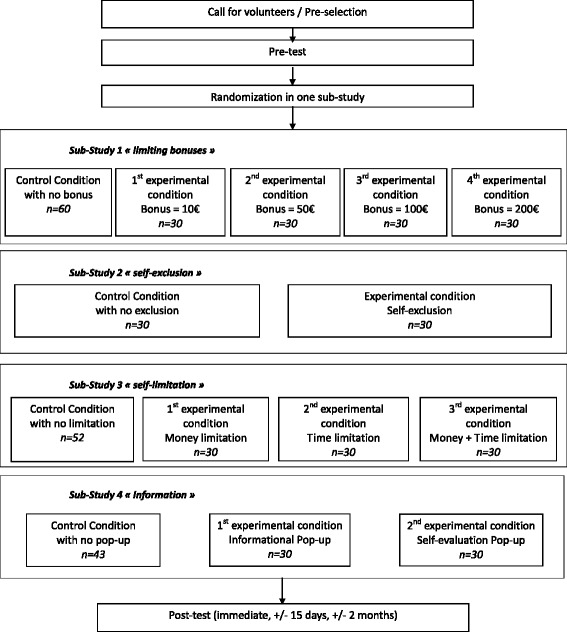
Study design

The participants are then randomly assigned to one experimental condition in one sub-study. Randomization is conducted according to two characteristics of each gambler: favorite game (games of chance without skill, semi-skilled games of chance, and skilled games) and gambler status (problem or non-problem). Each new participant included is randomized between the different experimental conditions and the control condition, based on these two characteristics in order to dispatch the gamblers among all the possibilities with equal number of gamblers having preference in each of the three major forms of games and equal number of problem or non-problem gamblers in each experimental condition. The number of participants to be included was computed according to the number of experimental conditions in each sub-study. In absence of anterior results, reliable power calculation and sample size determination are not possible. Thirty participants are assigned to each experimental condition and 30*√(k) are assigned to the control group of each sub-study (k being the number of experimental conditions in the sub-study) in order to optimize the comparison between each condition and the control group [18]. Consequently, each experimental condition and control groups is composed of an equal number of gamblers chosen among 6 possibilities crossing the three forms of game (games of chance without skill, semi-skilled games of chance, and skilled games) and the two possible gambling status (problem gambler or non-problem gambler). The final sample will consist of 485 volunteers (Fig. 1).

### Sub-study 1 “limiting bonuses”

The objective is to measure the effectiveness of limiting bonuses (currently nonexistent) and estimate the level of limitation for maximum efficiency. Efficiency will be measured according to the type of gambling and gambler status (problematic or not). A total of 180 participants will be recruited and randomized between the four proposed bonus amounts (€ 10 - n = 30, € 50 - n = 30, € 100 - n = 30, € 200 - n = 30) and the control group (n = 60), taking into account their game of choice and their gambling status.

The experimental session is a gambling situation on a computer at our research center. The gambler is invited to play on his favorite gambling site as usual, using his own gambling account and his own money. The participant is asked not to re-credit the account with any gains made during the experimental session (to avoid biasing measurement of the financial balance at the end of the gambling session). The presence of a camera will verify that the volunteer has not credited his in-game earnings (only the screen will be filmed). The gambling session ends when the participant wishes, but the session can last up to 4 h. The bonus given to the gambler is directly usable with a pre-paid card. It is given to the participant in the middle of the gambling session, to assess gambling behavior before and after obtaining the bonus.

An immediate post-test will be conducted at the end of the gambling session to collect the experimental data (Table 1).

**Table 1:** Data collected in the post-test for all the studies

Post-test	Measurements
Interview	Enjoyment during the gambling session
	Feeling out of control during the gambling session
	Moderator impact on gambling practice
	Interest of this moderator for themselves and/or for problem gamblers
	Look at the gambling operator who proposes this measure
Statement of gambling parameters	Balance of the gambler’s account during the game session (or since the last visit)
	Number of bets during the game session (or since the last visit)
	Money earned during the game session (or since the last visit)
	Gambling time during the game session (or since the last visit)
	Compliance by the participants with game predictions (ie amount of money or time that the gambler planned to spend in gambling before the game session)
Questionnaires	BMIS (mood assessment) [16]
	GRCS (cognitive distortions assessment) [15]
	CPGI (gambling problems assessment) [14]
	GACS (intensity of craving assessment) [17]

### Sub-study 2 “self-exclusion”

The objective is to measure the effectiveness of self-exclusion as a moderator of gambling practice. This will be assessed 15 days and 2 months after the self-exclusion procedure. This study includes only problem gamblers. In fact, this protection is not intended for non-problematic gamblers who control their practice.

In all, sixty participants will be recruited for this sub-study and will be randomly assigned to the control group (n = 30) or the experimental group (n = 30). To be included in the experimental group, the gambler must implement the measure of self-exclusion necessarily offered by gambling operators approved by ARJEL. The self-exclusion is performed via a pre-test with the help of the evaluator, and concerns only the site of their choice. The player is excluded for the next 7 days and has the choice, at the end of this period, to extend the ban or not.

A post-test interview is conducted by phone 15 days after the establishment of self-exclusion and another one 2 months later for the same purpose (Table 1).

### Sub-study 3 “self-limitation”

The objective is to define whether self-limitation is effective, and whether it is more effective in its present form, established by the ARJEL (limitation in terms of money wagered), in a different form (limitation of time spent in gambling) or in a form combining the two. A comparison of the effectiveness of self-limitation on the type of game and the gambler’s status is also performed. In all, 142 participants will be recruited and randomized between the three proposed limitations (money - n = 30, time - n = 30, money + time - n = 30) and the control group (n = 52), taking into account their game of choice and their gambler’s status.

The session starts with the same conditions as for the bonus sub-study. The gambling session ends when the gambler wishes, but not later than the time and money limits pre-determined during the pre-test. The session is blocked when the limits are reached.

An immediate post-test interview is conducted and another one by telephone 15 days after the experiment to get the same information (Table 1).

### Sub-study 4 “information”

The objective is to define whether pop-ups containing prevention messages are effective, and if these pop-up should contain informational messages (example: *“the outcome of the game depends only on chance”*), or self-evaluation messages (example: “*have you thought to check how long you play?*”). This study reproduces the methodology used by Blaszczynski and Monaghan in 2010 [12] on video lottery terminals.

A comparison of the effectiveness of these pop-ups depending on the game and the gambler’s status will also be performed. A total of 103 participants will be included and divided between the two experimental conditions (informational messages - n = 30, self-evaluation messages - n = 30) and the control group (n = 43), taking into account their game of choice and their gambler’s status. After that, the gambling session begins under the same conditions as for the other sub-studies. An immediate post-test interview is conducted and another one by telephone 15 days after the experiment to get the same information (Table 1).

## Discussion

The goal of this design is to evaluate the efficiency of the online gambling’s moderators proposed by French law and also to evaluate the efficiency of new ones, such as bonus limitation. Thus, at the end of this study, recommendations can be made to prevent gambling problems online. It is possible that some existing protections are less effective than expected, or can be improved. Improvements may be suggested to the existing moderator if the study records only low efficiency for it. Secondly, these recommendations would take into account the specificities of different types of games. In fact, gambling has different structural characteristics that affect the way you play. For example, one of our hypotheses is that the “time self-limitation” moderator will be more suitable for poker gamblers than for lottery gamblers. Indeed, playing poker can take a long time when participating in a tournament for example, while gambling the lottery can take only a few minutes. So we would be able to offer a special prevention program suitable for every type of game. Finally, our recommendations would take into account the particularities of the public concerned. Indeed, our study is designed to distinguish gamblers who keep control from those who lose control. Thus we would be able to offer moderators applicable to all gamblers, and more targeted actions for problem gamblers.

The major strength of this study is to put participants in a gambling situation at close to their normal conditions. So, participants play on real gambling sites (not simulators). Indeed, simulators only partially simulate real game situations: they don’t foster any interaction with other gamblers; they shorten the gambling sessions, etc. These characteristics certainly impact the motivation of the gamblers [19]. Moreover, our study design leads participants to gamble with real money. Conversely, simulators do not involve money bets, resulting in a modification of gambling behavior and lower risk taking [20, 21]. Finally, our participants gamble with their own money. In fact, gambling with money given by someone else influences gambling behavior, including lower risk-taking. The investment in the game (fighting, dipping, etc.) is lower if you have to defend your own money than if you can spend free money [19]. To complete that part on the strengths of the study, we can add that the very large sample size guarantees the validity of the results. In addition, taking into account the gambling type and gambler status allows us to obtain a population that reflects the diversity of gamblers, while most scientific studies do not take gambler heterogeneity into account.

The study has a few limitations. First of all, despite our best efforts, we were not able to build a completely satisfactory design. We tried to reproduce an experimental gambling situation as close as possible to the participants’ usual gambling situation. Nevertheless, the experiment takes place in a laboratory, and the gambling situation is obviously different from their usual surroundings. For example, the participants do not have the same sort of comfort (staying at home, playing on the couch, not being observed, etc.) and can’t link the gambling to other behaviors that influence practice (alcohol or other drugs associated…). We also have to deal with technical difficulties which may complicate recruitment. For example, self-limitation relative to money is already available on French gambling sites. So, it may be difficult to put bonus money on the gambler’s account if the participant has already self-limited. This is why we have decided to include in the bonus sub-study only participants with a betting limit of at least € 200 (to allow randomization under any experimental condition in this sub-study). Finally, for ethical reasons, we have chosen not to include pathological gamblers. The negative consequences in terms of lost money and loss of control during the experimental gambling session could be significant and have a negative impact on their gambling problem. In addition, we have assumed that the moderators considered in our study aim to prevent gambling problems, but are not intended for pathological gamblers who already encounter significant negative consequences associated with their gambling practice. They would further benefit from a brief Internet-based intervention on gambling websites and referral to a patient care setting.

Despite these limitations, this trial has many strengths which reinforce the importance of the results to prevent online gambling problem and maybe to influence policy-makers.

## Abbreviations

DSM-V: Diagnostic and Statistical Manual of Mental Disorders, Fifth Edition
ARJEL: Regulatory Authority for Online Gambling
EGMs: Electronic Gaming Machines
CPP: French Research Ethics Committee
CPGI: Canadian Problem Gambling Index
GRCS: Gambling Related Cognitions Scale
BMIS: Brief Mood Inspection Scale
GACS: Gambling Craving Scale
